# Partition decoupling for multi-gene analysis of gene expression profiling data

**DOI:** 10.1186/1471-2105-12-497

**Published:** 2011-12-30

**Authors:** Rosemary Braun, Gregory Leibon, Scott Pauls, Daniel Rockmore

**Affiliations:** 1 Department of Preventive Medicine and Robert H. Lurie Cancer Center, Northwestern University, Chicago, IL, USA; 2 National Cancer Institute, National Institutes of Health, Bethesda, MD, USA; 3 Department of Mathematics, Dartmouth College, Hanover, NH, USA; 4 Santa Fe Institute, Santa Fe, NM, USA

## Abstract

**Background:**

Multi-gene interactions likely play an important role in the development of complex phenotypes, and relationships between interacting genes pose a challenging statistical problem in microarray analysis, since the genes involved in these interactions may not exhibit marginal differential expression. As a result, it is necessary to develop tools that can identify sets of interacting genes that discriminate phenotypes without requiring that the classification boundary between phenotypes be convex.

**Results:**

We describe an extension and application of a new unsupervised statistical learning technique, known as the Partition Decoupling Method (PDM), to gene expression microarray data. This method may be used to classify samples based on multi-gene expression patterns and to identify pathways associated with phenotype, without relying upon the differential expression of individual genes. The PDM uses iterated spectral clustering and scrubbing steps, revealing at each iteration progressively finer structure in the geometry of the data. Because spectral clustering has the ability to discern clusters that are not linearly separable, it is able to articulate relationships between samples that would be missed by distance- and tree-based classifiers. After projecting the data onto the cluster centroids and computing the residuals ("scrubbing"), one can repeat the spectral clustering, revealing clusters that were not discernible in the first layer. These iterations, each of which provide a partition of the data that is decoupled from the others, are carried forward until the structure in the residuals is indistinguishable from noise, preventing over-fitting. We describe the PDM in detail and apply it to three publicly available cancer gene expression data sets. By applying the PDM on a pathway-by-pathway basis and identifying those pathways that permit unsupervised clustering of samples that match known sample characteristics, we show how the PDM may be used to find sets of mechanistically-related genes that may play a role in disease. An R package to carry out the PDM is available for download.

**Conclusions:**

We show that the PDM is a useful tool for the analysis of gene expression data from complex diseases, where phenotypes are not linearly separable and multi-gene effects are likely to play a role. Our results demonstrate that the PDM is able to distinguish cell types and treatments with higher accuracy than is obtained through other approaches, and that the Pathway-PDM application is a valuable technique for identifying disease-associated pathways.

## Background

Since their first use nearly fifteen years ago [1], microarray gene expression profiling experiments have become a ubiquitous tool in the study of disease. The vast number of gene transcripts assayed by modern microarrays (10^5^-10^6^) has driven forward our understanding of biological processes tremendously, elucidating the genes and regulatory mechanisms that drive specific phenotypes. However, the high-dimensional data produced in these experiments--often comprising many more variables than samples and subject to noise--also presents analytical challenges.

The analysis of gene expression data can be broadly grouped into two categories: the identification of differentially expressed genes (or gene-sets) between two or more known conditions, and the unsupervised identification (clustering) of samples or genes that exhibit similar profiles across the data set. In the former case, each gene is tested individually for association with the phenotype of interest, adjusting at the end for the vast number of genes probed. Pre-identified gene sets, such as those fulfilling a common biological function, may then be tested for an overabundance of differentially expressed genes (e.g., using gene set enrichment analysis [2]); this approach aids biological interpretability and improves the reproducibility of findings between microarray studies. In clustering, the hypothesis that functionally related genes and/or phenotypically similar samples will display correlated gene expression patterns motivates the search for groups of genes or samples with similar expression patterns. The most commonly used algorithms are hierarchical clustering [3], *k*-means clustering [4,5] and Self Organizing Maps [6]; a brief overview may be found in [7]. Of these, *k*-means appears to perform the best [7-9]. Relatedly, gene shaving [10] searches for clusters of genes showing both high variation across the samples and correlation across the genes, and several biclustering algorithms (such as [11]) search for class-conditional clusters of correlated genes. These methods are simple, visually appealing, and have identified a number of co-regulated genes and phenotype classes.

While these approaches have been fruitful, they also have the potential to miss causative mechanisms that can be deleteriously affected in several ways (such that no particular alteration is dominant in the case samples) as well as those which are only deleteriously affected through a combination of particular alterations (such that control samples may have some, but not all, the alterations necessary to produce the case phenotype). It is well known that complex diseases, such as cancers, exhibit considerable molecular heterogeneity for these reasons [12]. As a result, individual genes may fail to reach significance, and lists of differentially expressed genes or gene signatures may have poor concordance across studies. Additionally, pathway analyses that rely on single-gene association statistics (such as GSEA [2]) may fail to identify causative mechanisms. For the same reasons, clustering algorithms that rely on linearly-separable clusters (and hence upon differential expression between the clusters) may fail to partition the samples in a manner that reflects the true underlying biology.

As an example of how causative genes can be missed in gene-centric analyses, consider a recent study in which gene expression profiles in the Wagyu cattle are compared to those of the double-muscled Piedmontese cattle [13]. The Piedmontese cattle's muscular hypertrophy is attributable to a nonfunctional mutation of the myostatin gene (MSTN), but because MSTN itself is not differentially expressed between the two bovine models, its biological role cannot be inferred using traditional analyses of gene expression data. On the other hand, [13] showed that the functional MSTN variant was *co*-expressed with its regulatory target MYL2 in Wagyu cattle, whereas the nonfunctional variant in the Piedmontese cattle did not exhibit co-expression with MYL2. The correct identification of this system, in the absence of differential expression at the gene level in MSTN or MYL2, is crucial to understanding the molecular determinants of the double-muscled phenotype. This example serves to underscore the pressing need for analysis methods that can reveal *systems-level *differences in cases and controls even when the constituent genes do not exhibit differential expression.

As an alternative approach, we propose here an analysis technique that is designed to reveal relationships between samples based on multi-gene expression profiles without requiring that the genes be differentially expressed (i.e., without requiring the samples to be linearly separable in the gene-expression space), and that has the power to reveal relationships between samples at various scales, permitting the identification of phenotypic subtypes. Our approach adapts a new unsupervised machine-learning technique, the Partition Decoupling Method (PDM) [14,15], to gene expression data. The PDM consists of two iterated components: a spectral clustering step, in which the correlations between samples in the high-dimensional feature space are used to partition samples into clusters, followed by a scrubbing step, in which a projection of the data onto the cluster centroids is removed so that the residuals may be clustered. As part of the spectral clustering procedure, a low-dimensional nonlinear embedding of the data is used; as we will show in the Methods section, this both reduces the effect of noisy features and permits the partitioning of clusters with non-convex boundaries. The clustering and scrubbing steps are iterated until the residuals are indistinguishable from noise, as determined by comparison to a resampled null model. This procedure yields "layers" of clusters that articulate relationships between samples at progressively finer scales, and distinguishes the PDM from other clustering algorithms.

The PDM has a number of satisfying features. The use of spectral clustering allows identification of clusters that are not necessarily separable by linear surfaces, permitting the identification of complex relationships between samples. This means that clusters of samples can be identified even in situations where the genes do not exhibit differential expression, a trait that makes it particularly well-suited to examining gene expression profiles of complex diseases. The PDM employs a low-dimensional embedding of the feature space, reducing the effect of noise in microarray studies. Because the data itself is used to determine both the optimal number of clusters and the optimal dimensionality in which the feature space is represented, the PDM provides an entirely unsupervised method for classification without relying upon heuristics. Importantly, the use of a resampled null model to determine the optimal dimensionality and number of clusters prevents clustering when the geometric structure of the data is indistinguishable from chance. By scrubbing the data and repeating the clustering on the residuals, the PDM permits the resolution of relationships between samples at various scales; this is a particularly useful feature in the context of gene-expression analysis, as it permits the discovery of distinct sample subtypes. By applying the PDM to gene subsets defined by common pathways, we can use the PDM to identify gene subsets in which biologically meaningful topological structures exist, and infer that those pathways are related to the clinical characteristics of the samples (that is, if the genes in a particular pathway admit unsupervised PDM partitioning that corresponds to tumor/non-tumor cell types, one may infer that pathway's involvement in tumorigenesis). This pathway-based approach has the benefit of incorporating existing knowledge and being interpretable from a biological standpoint in a way that searching for sets of highly significant but mechanistically unrelated genes does not.

A number of other operationally similar, yet functionally distinct, methods have been considered in the literature. First, simple spectral clustering has been applied to gene expression data in [9], with mixed success. The PDM improves upon this both through the use of the resampled null model to provide a data-driven (rather than heuristic) choice of the clustering parameters, and by its ability to articulate independent partitions of the data (in contrast to a single layer) where such structure is present. As we will show, these aspects make the PDM more powerful than standard spectral clustering, yielding improved accuracy as well as the potential to identify sample subtypes that are not already known.

Another novel clustering method is proposed in [16], where an adaptive distance norm is used that can be shown to identify clusters of different shapes. The algorithm iteratively assigns clusters and refines the distance metric scaling parameter in a cluster-conditional fashion based on each cluster's geometry. This approach is able to identify clusters of mixed sizes and shapes that cannot be discriminated using fixed Euclidean or Mahalanobis distance metrics, and thus is a considerable improvement over *k*-means clustering. However, the method as described in [16] is computationally expensive and cannot identify non-convex clusters as spectral clustering, and hence the PDM, can.

Alternatively, SPACC [17] uses the same type of non-linear embedding of the data as is used in the PDM, which permits the articulation of non-convex boundaries. In SPACC [17], a single dimension of this embedding is used to recursively partition the data into two clusters. The partitioning is carried out until each cluster is solely comprised of one class of samples, yielding a classification tree. In this way, SPACC may also in some cases permit partitioning of known sample classes into subcategories. However, SPACC differs from the PDM in two crucial ways. First, the PDM's use of a data-determined number of informative dimensions permits more accurate clusterings than those obtained from a single dimension in SPACC. Second, SPACC is a semi-supervised algorithm that uses the known class labels to set a stopping threshold. Because there is no comparison to a null model, as in the PDM, SPACC will partition the data until the clusters are pure with respect to the class labels. This means that groups of samples with distinct molecular subtypes but identical class labels will remain unpartitioned (SPACC may not reveal novel subclasses) and that groups of samples with differing class labels but indistinguishable molecular characteristics will be artificially divided until the purity threshold is reached. By contrast, the clustering in the PDM does not impose assumptions about the number of classes or the relationship of the class labels to the clusters in the molecular data.

A fourth approach, QUBIC [11] is a graph theoretic algorithm that identifies sets of genes with similar class-conditional coexpression patterns (biclusters) by employing a network representation of the gene expression data and agglomeratively finding heavy subgraphs of co-expressed genes. In contrast to the unsupervised clustering of the PDM, QUBIC is a supervised method that is designed to find gene subsets with coexpression patterns that differ between pre-defined sample classes. In [11] it is shown that QUBIC is able to identify functionally related gene subsets with greater accuracy than competing biclustering methods; still, QUBIC is only able to identify biclusters in which the genes show strict correlation or anticorrelation coexpression patterns, which means that gene sets with more complex coexpression dynamics cannot be identified.

The PDM is thus unique in a number of ways: not only is it able to partition clusters with nonlinear and nonconvex boundaries, it does so in an unsupervised manner (permitting the identification of unknown subtypes) and in the context of comparison to a null distribution that both prevents clustering by chance and reduces the influence of noisy features. Moreover, the PDM's iterated clustering and scrubbing steps permit the identification of independent (i.e., decoupled) partitions in the data.

In this manuscript, we describe the PDM algorithm and demonstrate its application to several publicly-available gene-expression data sets. To illustrate the PDM's ability to articulate independent partitions of samples, we apply it to genome-wide expression data from a four phenotype, three exposure radiation response study [18]. The PDM partitions the samples by exposure and then by phenotype, yielding higher accuracy for predictions of radiation sensitivity than previously reported [18]. We also compare the PDM results to those obtained in a recent [9] comparison of clustering techniques, demonstrating the PDM's ability to identify cancer subtypes from global patterns in the gene expression data. Next, we apply the PDM using gene subsets defined by pathways rather than the global gene expression data, demonstrating how the PDM can be used to find biological mechanisms that relate to the phenotype of interest. We demonstrate Pathway-PDM in both the radiation response data [18] as well as a larger prostate cancer data set [19]. Our results suggest that the PDM is a powerful tool for articulating relationships between samples and for identifying pathways containing multi-gene expression patterns that distinguish phenotypes.

## Results and Discussion

### The Partition Decoupling Algorithm

The partition decoupling method (PDM) was first described in [14]. We summarize it here, and discuss its application to gene-expression data. The PDM consists of two iterated submethods: the first, spectral clustering, finds the dominant structures within the system, while the second "scrubbing" step removes this structure such that the next clustering iteration can distinguish finer-scale relationships within the residual data. The two steps are repeated until the residuals are indistinguishable from noise. By performing successive clustering steps, factors contributing to the partitioning of the data at different scales may be revealed.

#### Spectral Clustering

The first step, spectral clustering, serves to identify clusters of samples in high-dimensional gene-expression space. The motivation is simple: given a set of samples and a measure of pairwise similarity *s_ij _*between each pair, we wish to partition data in such a way that the samples within one cluster are significantly more similar to each other than they are to the remainder of the samples. A summary of the spectral clustering algorithm is given in Table 1.

**Table 1:** Procedure for Spectral Clustering.

	Spectral Clustering Algorithm
1.	Compute the correlation *ρ_ij _*between all pairs of *n *data points *i *and *j*.
2.	Form the similarity matrix **S **∈ ℝ^*n*×*n *^defined by *s_ij _*= exp [- sin^2 ^(arccos(*ρ_ij_*)/2)/*σ*^2^], where *σ *is a scaling parameter (*σ *= 1 in the reported results).
3.	Define **D **to be the diagonal matrix whose (i,i) elements are the column sums of **S**.
4.	Define the Laplacian **L **= **I **- **D**^-1/2^**SD**^-1/2^.
5.	Find the eigenvectors {*v*_0_, *v*_1_, *v*_2_, . . . , *v*_*n*-1_} with corresponding eigenvalues 0 ≤ *λ*_1 _≤ *λ*_2 _≤ ⋯ ≤ *λ*_*n*-1 _of **L**.
6.	Determine from the eigendecomposition the optimal dimensionality *l *and natural number of clusters *k *(see text).
7.	Construct the embedded data by using the first *l *eigenvectors to provide coordinates for the data (i.e., sample *i *is assigned to the point in the Laplacian eigenspace with coordinates given by the *i*th entries of each of the first *l *eigenvectors, similar to PCA).
8.	Using *k*-means, cluster the *l*-dimensional embedded data into *k *clusters.

Spectral clustering offers several advantages over traditional clustering algorithms such as those reviewed in [7]. Most importantly, no constraint is placed on the geometry of the data, in contrast to the tree-like structure imposed by hierarchical clustering [3] or the necessity of convexity of the clusters for detection via distance-based *k*-means clustering as used in [4,5], and in Self Organizing Maps [6]. Spectral clustering also uses a low-dimensional embedding of the data, thus excluding the noisy, high-frequency components.

In spectral clustering, the data are represented as a complete graph in which nodes correspond to samples and edge weights *s_ij _*correspond to some measure of similarity between a pair of nodes *i *and *j*. Spectral graph theory (see, e.g., [20]) is brought to bear to find groups of connected, high-weight edges that define clusters of samples. This problem may be reformulated as a form of the min-cut problem: cutting the graph across edges with low weights, so as to generate several subgraphs for which the similarity between nodes is high and the cluster sizes preserve some form of balance in the network. It has been demonstrated [20-22] that solutions to relaxations of these kinds of combinatorial problems (i.e., converting the problem of finding a minimal configuration over a very large collection of discrete samples to achieving an approximation via the solution to a related continuous problem) can be framed as an eigendecomposition of a graph Laplacian matrix **L**. The Laplacian is derived from the similarity matrix **S **(with entries *s_ij_*) and the diagonal degree matrix **D **(where the *i*th element on the diagonal is the degree of entity *i*, ∑*_j _s_ij_*), normalized according to the formula

(1) $$L\text{ = }L-D^{-1∕2}SD^{-1∕2}.$$

In spectral clustering, the similarity measure *s_ij _*is computed from the pairwise distances *r_ij _*between samples *i *and *j *using a Gaussian kernel [20-22] to model local neighborhoods,

(2) $$s_{ij}=\exp\left(\frac{-r_{ij}^{2}}{2\sigma^{2}}\right),$$

where scaling the parameter *σ *controls the width of the Gaussian neighborhood, i.e., the scale at which distances are deemed to be similar. (In our analysis, we use *σ *= 1, though it should be noted that how to optimally choose *σ *is an open question [21,22].) Following [15], we use a correlation-based distance metric in which the correlation *ρ_ij _*between samples *i *and *j *is converted to a chord distance on the unit sphere,

(3) $$r_{ij}=2\sin\left(\arccos\left(\rho_{ij}\right)∕2\right).$$

The use of the signed correlation coefficient implies that samples with strongly anticorrelated gene expression profiles will be dissimilar (small *s_ij_*) and is motivated by the desire to distinguish between samples that positively activate a pathway from those that down-regulate it.

Eigendecomposition of the normalized Laplacian **L **given in Eq. 1 yields a spectrum containing information regarding the graph connectivity. Specifically, the number of zero eigenvalues corresponds to the number of connected components. In the case of a single connected component (as is the case for almost any correlation network), the eigenvector for the second smallest (and thus, first nonzero) eigenvalue (the normalized Fiedler value *λ*_1 _and Fiedler vector *v*_1_) encodes a coarse geometry of the data, in which the coordinates of the normalized Fiedler vector provide a one-dimensional embedding of the network. This is a "best" embedding in the sense that it solves a relaxation of an optimization problem that seeks to determine an optimal partitioning of the data (see [20-22]). This one-dimensional summary provides the greatest dimension reduction--but optimal with respect to the dimensionality--of the data. Finer resolution is provided by the dimension reductions obtained by increasing the dimensionality via the use of additional eigenvectors (in order, according to increasing eigenvalue).

By embedding the data into a smaller-dimensional space defined by the low-frequency eigenvectors and clustering the embedded data using *k*-means [4], the geometry of the data may be revealed. Because *k*-means clustering is by nature stochastic [4], multiple *k*-means runs are performed and the clustering yielding the smallest within-cluster sum of squares is chosen. In order to use *k*-means on the embedded data, two parameters need to be chosen: the number of eigenvectors *l *to use (that is, the dimensionality of the embedded data) and the number of clusters *k *into which the data will be clustered.

##### Optimization of *l*

The optimal dimensionality of the embedded data is obtained by comparing the eigenvalues of the Laplacian to the distribution of Fiedler values expected from null data. The motivation of this approach follows from the observation that the size of eigenvalues corresponds to the degree of structure (see [22]), with smaller eigenvalues corresponding to greater structure. Specifically, we wish to construct a distribution of null Fiedler values--eigenvalues encoding the coarsest geometry of randomly organized data--and select the eigenvalues from the true data that are significantly small with respect to this distribution (below the 0.05 quantile). In doing so, we select the eigenvalues that indicate greater structure than would be expected by chance alone. The idea is that the distribution of random Fiedler values give a sense of how much structure we could expect of a comparable random network. We thus take a collection of perpendicular axes, onto each of which the projection of the data would reveal more structure than we would expect at random.

The null distribution of Fiedler values is obtained through resampling *s_ij _*(preserving *s_ij _*= *s_ji _*and *s_ii _*= 1). This process may be thought of as "rewiring" the network while retaining the same distribution of edge weights. This has the effect of destroying structure by dispersing clusters (subgraphs containing high edge weights) and creating new clusters by random chance. Because the raw data itself is not resampled, the resulting resampled network is one which has the same marginal gene expression distributions and gene-gene correlations as the original data, and is thus a biologically comparable network to that in the true data. Note that the resampling-based (and hence nonparametric) construction of the reference distribution here differs from the previous description of the PDM [15] that employed a Gaussian ensemble null model.

Eigenvectors whose eigenvalues are significantly small with respect to the resampled null model are retained as the coordinates that describe the geometry of the system that distinguishable from noise, yielding a low-dimensional embedding of the significant geometry. If none of the eigenvalues are significant with respect to the resampled null reference distribution, we conclude that no coordinate encodes more significant cluster structure than would be obtained by chance, and halt the process.

##### Optimization of *k*

The development of methods for obtaining the number of clusters *k *suitable for partitioning a data set is an area of active research (see, e.g., [22,23] and references therein). Our approach exploits the property [15,22] that clustering the entries in the Fiedler vector yields the best decomposition of the network components. Consequently, one can use the number peaks in the density of the Fiedler vector--that is, the number of values about which the elements of *v*_1 _are clustered--as the number of clusters. (This procedure is roughly analogous to finding regions of high density along the first principle component of the data.) To obtain this value, we fit a Gaussian mixture model [24] with 2-30 components (assuming unequal variances), compute the Bayesian Information Criterion (BIC) for each mixture model, and choose the optimum number of components (for details of the implementation, see [25,26]).

Once *k *and *l *have been assigned, the data embedded via the coordinates given by the eigenvectors of the smallest *l *nonzero eigenvalues is clustered using *k*-means [4]. The spectral clustering procedure offers several advantages over simple clustering of the original data using *k*-means: first, the Fiedler vector provides a natural means to estimate the number of clusters; and second, because spectral clustering operates on similarity of the samples, rather than planar cuts of the high-dimensional feature space, complex correlation structures can be identified. A complete discussion of the advantages of spectral clustering is given in [20-22].

**Figure 1:**
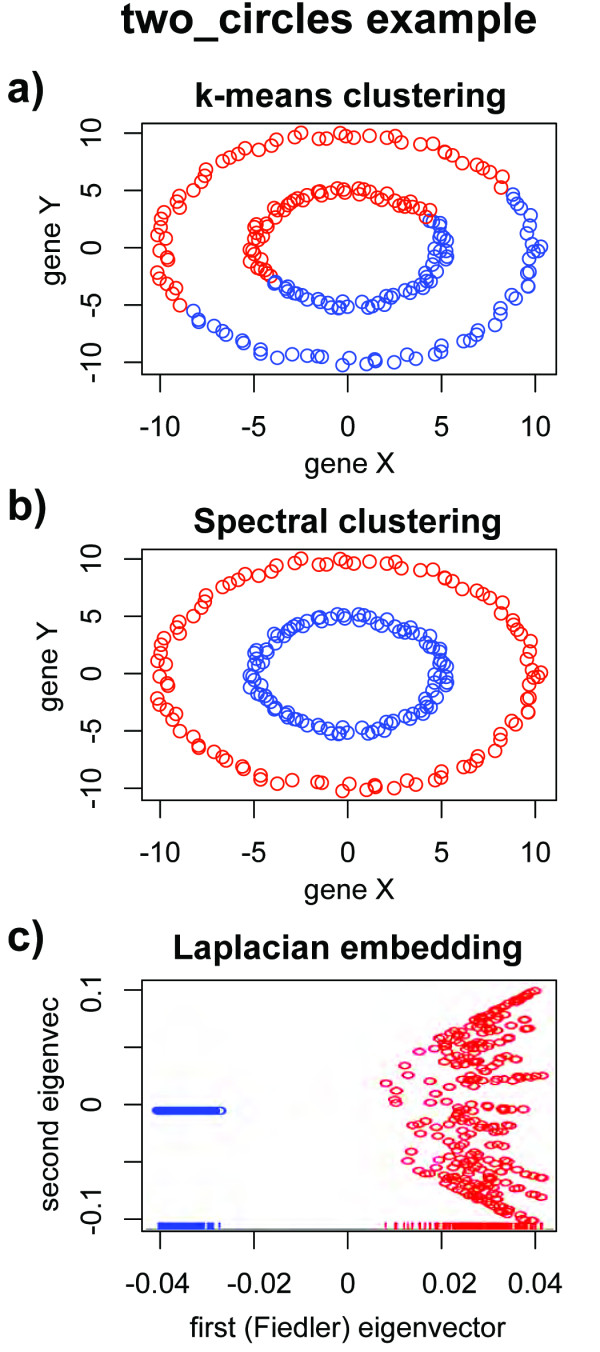
**two circles examples**. In (a) and (b), colors denote cluster assignments from *k*-means (*k *= 2) and spectral clustering, respectively. In (a), *k*-means using *k *= 2 produces a linear cut through the data; in (b), spectral clustering automatically chooses two clusters and assigns clusters with nonconvex boundaries. The spectrally embedded data used in (b) is shown in (c); in this representation, the clusters are linearly separable, and a rug plot shows the bimodal density of the Fiedler vector that yielded the correct number of clusters.

To illustrate the power of this method, consider a toy data set called "two_circles" in which 200 data points are placed in two-dimensional space in two concentric circles, as depicted in Figure 1. Because *k*-means alone can only identify clusters with convex hulls, *k*-means clustering using *k *= 2 produces an arbitrary, linear division of the data as shown in Figure 1(a). In contrast, spectral clustering identifies the two rings as individual clusters, as seen in Figure 1(b). While *k*-means took *k *= 2 as an input from the user, the spectral clustering example determined *k *= 2 from the data, as shown in Figure 1(c); the rug plot depicts the distribution of the Fiedler vector coordinates, in which two peaks are readily visible and chosen as indicative of two clusters, as described above.

**Figure 2:**
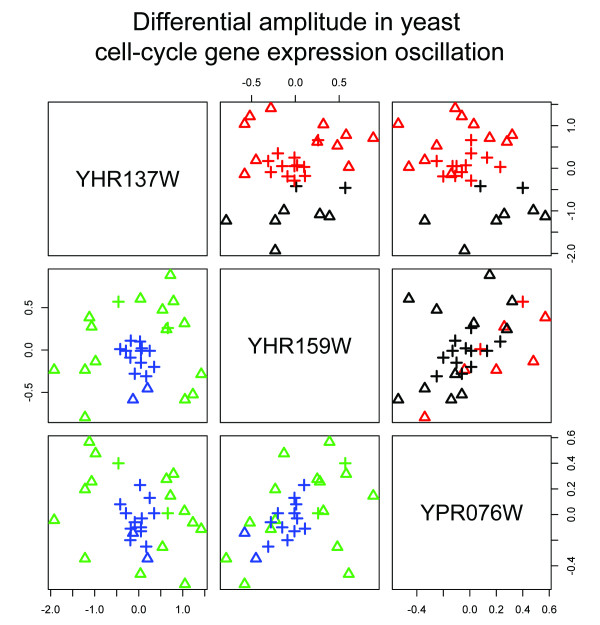
**Yeast cell cycle data**. Expression levels for three oscillatory genes are shown. The method of cell cycle synchronization is shown as shapes: crosses denote elutriation-synchronized samples, while triangles denote CDC-28 synchronized samples. Cluster assignment for each sample is shown by color; above the diagonal, points are colored by *k*-means clustering, with poor correspondence between cluster (color) and synchronization protocol (shapes); below the diagonal, samples are colored by spectral clustering assignment, showing clusters that correspond to the synchronization protocol.

While the two_circles data is simulated, we note that patterns of this type will arise when out-of-phase oscillatory genes, such as those involved in circadian rhythms or cell cycle processes, are sampled; the radii of the co-expression circles will be dictated by the amplitude of the gene oscillations. An illustration of such patterns in nature is provided in Figure 2, which depicts the co-expression pattern of three cell-cycle related genes in CDC-28 and elutriation synchronized yeast cells from [27]. The elutriation synchronized cells exhibit much smaller amplitude oscillations than do the CDC-28 synchronized cells; while the CDC-28 and elutriation synchronized cells cannot be distinguished using k-means, the distinction is readily made via spectral clustering. The biological relevance of patterns such as those depicted in Figures 1 and 2 has been noted in mammalian systems as well; in [28] it is found that the majority of mammalian genes oscillate and that the amplitude of oscillatory genes differs between tissue types and is associated with the gene's function. These observations led to the conclusion in [28] that pathways should be considered as dynamic systems of genes oscillating in coordination with each other, and underscores the need to detect amplitude differences in co-oscillatory genes as depicted in Figures 1 and 2.

The benefit of spectral clustering for pathway-based analysis in comparison to over-representation analyses such as GSEA [2] is also evident from the two_circles example in Figure 1. Let us consider a situation in which the *x*-axis represents the expression level of one gene, and the *y*-axis represents another; let us further assume that the inner ring is known to correspond to samples of one phenotype, and the outer ring to another. A situation of this type may arise from differential misregulation of the *x *and *y *axis genes. However, while the variance in the *x*-axis gene differs between the "inner" and "outer" phenotype, the means are the same (0 in this example); likewise for the *y*-axis gene. In the typical single-gene *t*-test analysis of this example data, we would conclude that neither the *x*-axis nor the *y*-axis gene was differentially expressed; if our gene set consisted of the *x*-axis and *y*-axis gene together, it would not appear as significant in GSEA [2], which measures an abundance of single-gene associations. Yet, unsupervised spectral clustering of the data would produce categories that correlate exactly with the phenotype, and from this we would conclude that a gene set consisting of the *x*-axis and *y*-axis genes plays a role in the phenotypes of interest. We exploit this property in applying the PDM by pathway to discover gene sets that permit the accurate classification of samples.

#### Scrubbing

After the clustering step has been performed and each data point assigned to a cluster, we wish to "scrub out" the portion of the data explained by those clusters and consider the remaining variation. This is done by computing first the cluster centroids (that is, the mean of all the datapoints assigned to a given cluster), and then subtracting the data's projection onto each of the centroids from the data itself, yielding the residuals. The clustering step may then be repeated on the residual data, revealing structure that may exist at multiple levels, until either a) no eigenvalues of the Laplacian in the scrubbed data are significant with resepct to those obtained from the resampled graphs as described above; or b) the cluster centroids are linearly dependent. (It should be noted here that the residuals may still be computed in the latter case, but it is unclear how to interpret linearly dependent centroids.)

### Application to Microarray Data

We apply the PDM to several cancer gene expression data sets to demonstrate how it may be used to reveal multiple layers of structure. In the first data set [18], the PDM articulates two independent partitions corresponding to cell type and cell exposure, respectively. Analysis of the second data [9] set demonstrates how successive partitioning by the PDM can reveal disease and tissue subtypes in an unsupervised way. We then show how the PDM can be used to identify the biological mechanisms that drive phenotype-associated partitions, an approach that we call "Pathway-PDM." In addition to applying it to the radiation response data set mentioned above [18], we also apply Pathway-PDM to a prostate cancer data set [19], and briefly discuss how the Pathway-PDM results show improved concordance of significant pathways identified in the Singh data [19] with those previously identified in several other prostate cancer data sets [29].

### Partition Decoupling in Cancer Gene Expression Data

#### Radiation Response Data

**Figure 3:**
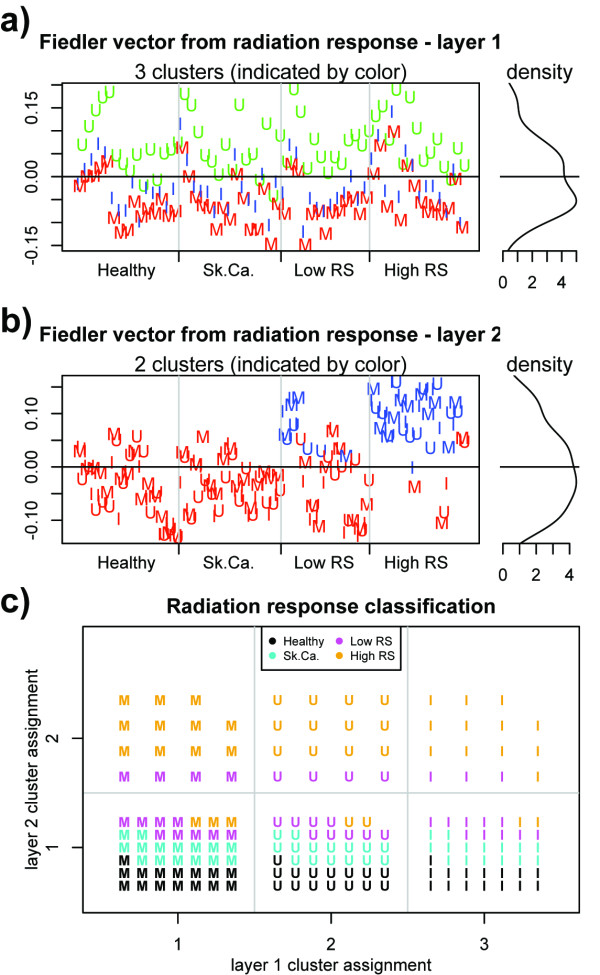
**PDM results for radiation response data**. In (a) and (b) we see scatter plots of each sample's Fiedler vector value along with the resulting clustering (indicated by color) for the first (a) and second (b) PDM layers. A Gaussian mixture fit to the density (left panel) of the Fiedler vector is used to assess the number of clusters, and the resulting cluster assignment for each sample is indicated by color. Exposure is indicated by shape ("M"-mock; "U"-UV; "I"-IR), with phenotypes (healthy, skin cancer, radiation insensitive, radiation sensitive) grouped together along the *x*-axis. In (a), it can be seen that the cluster assignment correlates with exposure, while in (b), cluster assignment correlates with radiation sensitivity. In (c), points are placed in the grid according to cluster assignment from layers 1 and 2 along the *x *and *y *axes; it can be seen that the UV-and IR- exposed high-sensitivity samples differ both from the mock-exposed high-sensitivity samples as well as the UV- and IR- exposed control samples.

We begin by applying the PDM to the radiation response data [18] to illustrate how it may be used to reveal multiple layers of structure that, in this case, correspond to radiation exposure and sensitivity. In the first layer, spectral clustering classifies the samples into three groups that correspond precisely to the treatment type. The number of clusters was obtained using the BIC optimization method as described above. Resampling of the correlation coefficients was used to determine the dimension of the embedding *l *using 60 permutations (increasing this further did not alter the eigenvalues deemed significant); 30 *k*-means runs were performed and the clustering yielding the smallest within-cluster sum of squares was chosen. Classification results are given in Table 2 and Figure 3(a). The unsupervised algorithm correctly identifies that three clusters are present in the data, and assigns samples to clusters in a manner consistent with their exposure.

**Table 2:** Spectral clustering of expression data versus exposure; exposure categories are reproduced exactly.

	Cluster		
	
	1	2	3
Mock	57	0	0
IR	0	57	0
UV	0	0	57

In order to compare the performance of spectral clustering to that of *k*-means, we ran *k*-means on the original data using *k *= 3 and *k *= 4, corresponding to the number of treatment groups and number of cell type groups respectively. As with the spectral clustering, 30 random *k *means starts were used, and the smallest within-cluster sum of squares was chosen. The results, given in Tables 3 and 4, show substantially noisier classification than the results obtained via spectral clustering. It should also be noted that the number of clusters *k *used here was not derived from the characteristics of the data, but rather is assigned in a supervised way based on additional knowledge of the probable number of categories (here, dictated by the study design). While the pure *k*-means results are noisy, the *k *= 4 classification yields a cluster that is dominated by the highly radiation-sensitive cells (cluster 4, Table 4). Membership in this cluster versus all others identifies highly radiation-sensitive cells with 62% sensitivity and 96% specificity; if we restrict the analysis to the clinically-relevant comparison between the last two cell types--that is, cells from cancer patients who show little to no radiation sensitivity and those from cancer patients who show high radiation sensitivity--the classification identifies radiation-sensitive cells with 62% sensitivity and 82% specificity.

**Table 3:** *k*-means clustering of expression data versus exposure using *k *= 3.

	Cluster		
	
	1	2	3
Mock	36	15	6
IR	36	15	6
UV	3	14	40

**Table 4:** *k*-means clustering of expression data versus cell type using *k *= 4.

	Cluster			
	
	1	2	3	4
Healthy	19	18	8	0
Skin cancer	8	23	14	0
Low radiation sensitivity	13	11	8	7
High radiation sensitivity	6	1	9	26

The result from the *k *= 4 *k*-means classification suggest that there exist cell-type specific differences in gene expression between the high radiation sensitivity cells and the others. To investigate this, we perform the "scrubbing" step of the PDM, taking only the residuals of the data after projecting onto the clusters obtained in the first pass. As in the first layer, we use the BIC optimization method to determine the number of clusters *k *and resampling of the correlations to determine the dimension of the embedding *l *using 60 permutations. The second layer of structure revealed by the PDM partitioned the high-sensitivity samples from the others into two clusters. Classification results are given in Table 5 and Figure 3(b), and it can be seen that the partitioning of the radiation-sensitive samples is highly accurate (83% sensitivity and 91% specificity across all samples).

**Table 5:** Spectral clustering of exposure data with exposure-correlated clusters scrubbed out, versus cell type.

	Cluster	
	
	1	2
Healthy	45	0
Skin cancer	45	0
Low radiation sensitivity	28	11
High radiation sensitivity	7	35

Further PDM iterations resulted in residuals that were indistinguishable from noise (see Methods); we thus conclude that there are only two layers of structure present in the data: the first corresponding to exposure, and the second to radiation sensitivity. That is, there exist patterns in the gene expression space that distinguish UV- and ionizing radiation exposed cells from mock-treated cells (and from each other), and that there exist further patterns that distinguish high-sensitivity cells from the rest. Together, these independent (decoupled) sets of clusters describe six categories, as shown in Figure 3(c), wherein the second layer partitions the radiation sensitive cells from the others in each exposure-related partition. The fact that the mock-exposure as well as the UV- and IR-exposure partitions are further divided by radiation sensitivity in the second layer suggests that there exist constitutive differences in the radiation sensitive cells that distinguish them from the other groups even in the absence of exposure. Importantly, the data-driven methodology of the PDM identifies only phenotypic clusters, corresponding to the high-sensitivity cells and the three control groups combined, without further subpartitioning the combined controls. This suggests that the three control groups do not exhibit significant differences in their global gene-expression profiles.

In the original analysis of this data [18], the authors used a linear, supervised algorithm (SAM, a nearest shrunken centroids classifier [30]) to develop a predictor for the high-sensitivity samples. This approach obtained 64.2% sensitivity and 100% specificity [18], yielding a clinically useful predictor. The PDM's unsupervised detection of the high sensitivity sample cluster suggests that the accuracy in [18] was not a result of overfitting to training data; moreover, the PDM's ability to identify those samples with higher sensitivity than in [18] indicates that there exist patterns of gene expression distinct to the radiation-sensitive patients which were not identified in the SAM analysis, but are detectable using the PDM.

#### DeSouto Multi-study Benchmark Data

Having observed the PDM's ability to decouple independent partitions in the four-phenotype, three-exposure radiation response data, we next consider the PDM's ability to articulate disease subtypes. Because cancers can be molecularly heterogeneous, it is often important to articulate differences between subtypes--a distinction that may be more subtle than than the differences caused by radiation exposure. Here, we apply the PDM to the suite of 21 Affymetrix data sets previously considered in [9]. The use of these sets is motivated by their diversity and by the ability to compare the PDM performance to that of the methods reported in [9].

In [9], the authors applied several widely used clustering algorithms--spectral clustering, hierarchical clustering, *k*-means, finite mixture of Gaussians (FMG), and shared nearest-neighbor clustering--to the data using various linkage and distance metrics as available for each. In [9], the number of clusters *k *was set manually, ranging over $\left(k_{c},\sqrt{n}\right)$, where *k_c _*is the known number of sample classes and *n *is the number of samples; in the spectral clustering implementation, *l *was set equal to the value chosen for *k*. Note that the PDM differs in several crucial ways from basic spectral clustering as applied in [9]. First, the choices of *k *and *l *in the PDM are data-driven (thus allowing a priori values for *k *that is smaller than *k_c_*, and as many dimensions *l *as are significant compared to the null model as previously described). Second, the successive partitioning carried out in the PDM layers can disambiguate mixed clusters. Notably, the PDM partitions are obtained without relying on prior knowledge of the number of clusters. This is an important feature when the data may contain un-identified disease subtypes.

**Figure 4:**
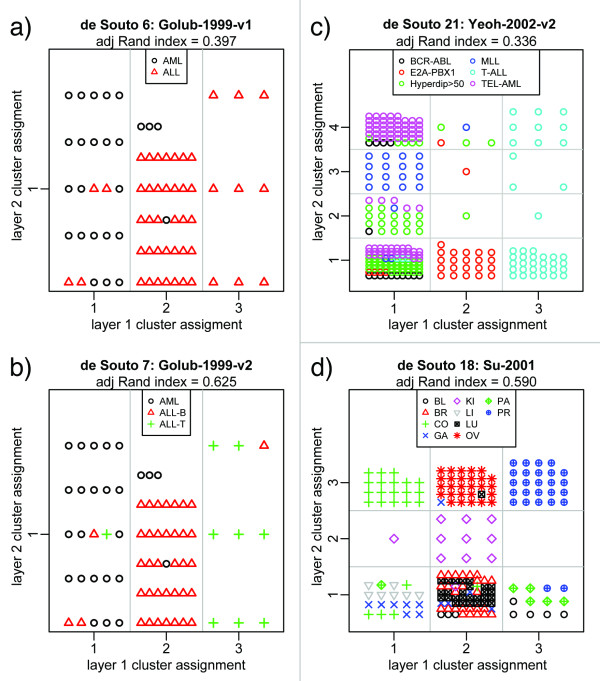
**PDM results for several benchmark data sets**. Points are placed in the grid according to cluster assignment from layers 1 and 2 (in (a) and (b) no second layer is present). In (a) and (b) it can be seen that the PDM identifies three clusters, and that the division of the ALL samples in (a) corresponds to a subtype difference (ALL-B, ALL-T) shown in (b). In (c) and (d), it can be seen that the partitioning of samples in the first layer is refined in the second PDM layer.

To illustrate this, we focus on a handful of the benchmark data sets. (Full results are provided in Additional Files 1 and 2.) The partitions are shown in Figure 4. In Figure 4(a) and 4(b), PDM reveals a single layer of three clusters in two versions of the Golub-1999 leukemia data [31]. The two data sets as provided contained identical gene expression measurements and differed only in the sample status labels, with Golub-1999-v1 only distinguishing AML from ALL, but Golub-1999-v2 further distinguishing between B- and T-cell ALL. As can be seen from Figure 4(a,b), the PDM articulates a single layer of three clusters, based on the gene expression data. In Figure 4(a) (Golub-1999-v1), we see that the AML samples are segregated into cluster 1, while the ALL samples are divided amongst clusters 2 and 3; that is, the PDM partition indicates that there exists structure, distinct from noise (as defined through the resampled null model), that distinguishes the ALL samples as two subtypes. If we repeat this analysis with Golub-1999-v2, we obtain the partitions shown in Figure 4(b). Since the actual gene expression data is identical, the PDM partitioning of samples is the same; however, we now can see that the division of the ALL samples between clusters 2 and 3 corresponds to the B- and T-cell subtypes. One can readily find--particularly in the context of cancers--situations in which unknown sample subclasses exist that could be detected via PDM (as in Figure 4(a)); at the same time, the PDM's comparison to the resampled null model prevents artificial partitions of the data.

In Figures 4(c) and 4(d), we see how the first layer of clustering is refined in the second layer; for example, in Figure 4(c), the E2A-PBX1 and T-ALL leukemias are distinguished in the first layer, while the second serves to separate the MLL and majority of the TEL-AML subtypes from the mixture of B-cell ALLs in the first cluster of layer 1. As in Figures 4(a) and 4(b), the PDM identifies clusters of subtypes that may not be known a priori (cf. results for Yeoh-2002-v1 in Additional Files 1 and 2, for which all the B-cell ALLs had the same class label but were partitioned, as in Figure 4(c), by several subtypes). In Figure 4(d), second layer cluster assignment in Figure 4(d) distinguishes the ovarian (OV) and kidney (KI) samples from the others in the mixed cluster 2 in the first layer.

Results for the complete set of Affymetrix benchmark data are given in Additional Files 1 and 2. A *t*-test comparison of adjusted Rand indices obtained from the PDM suggests that it is comparable to those obtained with the best method, FMG, in [9]. However, it is important to note that this is achieved by the PDM in an entirely unsupervised way (in contrast to the heuristic approach used to select *k *and *l *in [9]). This is a considerable advantage. We also note that the PDM performance remained high regardless of the distance metric used (cf. Fig. S-1 vs. Fig. S-2 in Additional Files 1 and 2), and we did not observe the large decrease in accuracy noted by [9] when using a Euclidean metric in spectral clustering. We attribute this largely to the aforemented improvements (multiple layers; data-driven *k *and *l *parameterization) of the PDM over standard spectral clustering.

### Pathway-PDM Analysis

The above applications of the PDM illustrate its ability to detect clusters of samples with common exposures and phenotypes based on genome-wide expression patterns, without advance knowledge of the number of sample categories. However, it is often of greater interest to identify a set of genes that govern the distinction between samples. Pathway-based application of the PDM permits this by systematically subsetting the genes in known pathways (here, based on KEGG [32] annotations), and partitioning the samples. Pathways yielding cluster assignments that correspond to sample characteristics can then be inferred to be associated with that characteristic. We call this approach the "Pathway-PDM."

**Figure 5:**
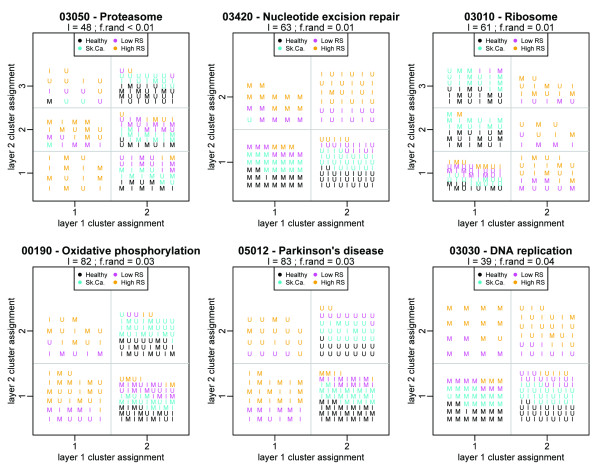
**Pathway-PDM results for top pathways in radiation response data**. Points are placed in the grid according to cluster assignment from layers 1 and 2 along for pathways with *f*_rand _*<*0.05. Exposure is indicated by shape ("M"-mock; "U"-UV; "I"-IR), with phenotypes (healthy, skin cancer, low RS, high RS) indicated by color. Several pathways (nucleotide excision repair, Parkinson's disease, and DNA replication) cluster samples by exposure in one layer and phenotype in the other, suggesting that these mechanisms differ between the case and control groups.

We applied Pathway-PDM as described above to the radiation response data from [18], testing the clustering results obtained for inhomogeneity with respect to the phenotype (*χ*^2 ^test). Because some pathways contain a fairly large number of probes, it is reasonable to ask whether the pathways that permitted clusterings corresponding to tumor status were simply sampling the overall gene expression space. In order to assess this, we also constructed artificial pathways of the same size as each real pathway by randomly selecting the appropriate number of probes, and recomputing the clustering and *χ*^2 ^*p*-value as described above. 1000 such random pathways were created for each unique pathway length, and the fraction *f*_rand _of pathways that yielded a *χ*^2 ^*p*-value smaller than that observed in the "true" pathway is used as an additional measure of the pathway significance. Six pathways distinguished the radiation-sensitive samples with *f*_rand _*<*0.05 as shown in Figure 5; several also articulated exposure-associated partitions in addition to the phenotype-associated partition. Interestingly, all of the high-scoring pathways separated the high-RS case samples, but did not subdivide the three control sample classes; this finding, as well as the exposure-independent clustering assignments in several pathways in Figure 5, suggests that there are systematic gene expression differences between the radiation-sensitive patients and all others. Several other pathways (see Figure S-3 in Additional File 3) yield exposure-associated partitions without distinguishing between phenotypes; unsurprisingly, these are the cell cycle, p53 signaling, base excision repair, purine metabolism, MAP kinase, and apoptosis pathways.

To further illustrate Pathway-PDM, we apply it to the Singh prostate gene expression data [19] (the heavily-filtered sets from [9] have too few remaining probes to meaningfully subset by pathway). First, we observe that in the complete gene expression space, the clustering of samples corresponds to the tumor status in the second PDM layer (Figure S-4 in Additional File 4). This is consistent with the molecular heterogeneity of prostate cancer, and suggests that the first layer describes individual variation that is scrubbed out and then revealed in the second layer. Next, we apply Pathway-PDM as described above, testing each layer of clustering for inhomogeneity with respect to the known tumor/normal labels (*χ*^2 ^test).

**Figure 6:**
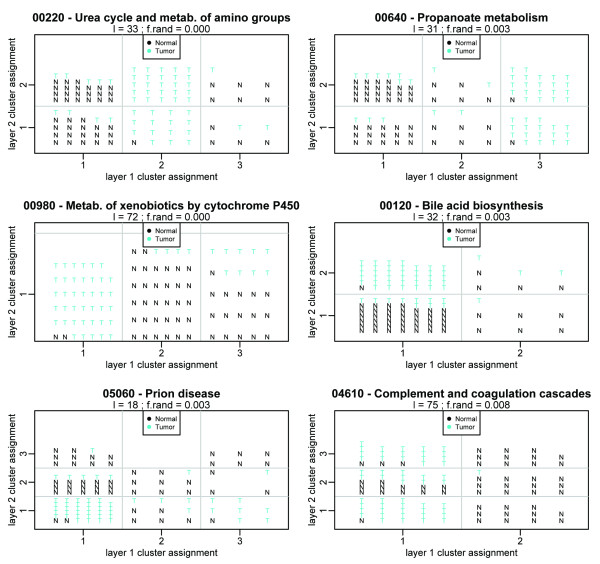
**Pathway-PDM results for the six most discriminative pathways in the Singh prostate data**. Points are placed in the grid according to cluster assignment from layers 1 and 2.

Of the 203 pathways considered, those that yielded significant *f*_rand _in any layer of clustering is given in Table 6. No pathway yielded more than two layers of structure. A total of 29 of 203 pathways exhibited significant clustering inhomogeneity in any layer; amongst the significant pathways, the misclassification rate--the fraction of tumor samples that are placed in a cluster that is majority non-tumor and vice-versa--is approximately 20%. Plots of the six most discriminative pathways in layers 1 and 2 are given in Figure 6.

**Table 6:** Pathways with cluster assignment articulating tumor versus normal status in at least one PDM layer for the Singh prostate data.

			Layer 1		Layer 2		
					
	KEGG Pathway	*L_p_*	*p *(*χ*^2^)	*f*_rand_	*p *(*χ*^2^)	*f*_rand_	**In **[29]** ?**
00220	Urea cycle & metabolism of amino groups	33	1.14e-13	*<*0.001	7.10e-01	0.940	[19,38,39]
00980	Metab. of xenobiotics by cytochrome P450	72	3.97e-13	0.001			--
00640	Propanoate metabolism	31	7.78e-12	0.003	9.78e-01	0.995	[38,39]
04610	Complement and coagulation cascades	75	9.21e-12	0.008	2.47e-02	0.371	
00120	Bile acid biosynthesis	32	1.29e-01	0.699	1.15e-11	0.003	[19,38]
05060	Prion disease	18	5.18e-02	0.527	2.20e-11	0.003	[19,38,39]
00380	Tryptophan metabolism	50	3.84e-11	0.008	5.52e-01	0.894	[39]
00480	Glutathione metabolism	48	4.80e-11	0.008	8.37e-01	0.955	[19,38,39]
04310	Wnt signaling pathway	191	5.38e-11	0.017	5.47e-01	0.916	[38]
00983	Drug metabolism - other enzymes	52	5.08e-10	0.024	8.60e-01	0.966	--
04630	Jak-STAT signaling pathway	205	1.65e-01	0.826	8.41e-10	0.025	
00053	Ascorbate and aldarate metabolism	8	3.32e-02	0.462	7.67e-09	0.008	[39]
00350	Tyrosine metabolism	45	1.32e-02	0.359	2.80e-08	0.040	[19,38]
00641	3-Chloroacrylic acid degradation	16	5.23e-08	0.016	6.89e-01	0.893	
00960	Alkaloid biosynthesis II	8	7.13e-02	0.558	8.23e-08	0.016	[19]
00410	beta-Alanine metabolism	25	9.24e-08	0.016	1.60e-01	0.673	[39]
00650	Butanoate metabolism	37	9.39e-02	0.645	1.50e-07	0.014	
00260	Glycine, serine & threonine metabolism	36	9.56e-02	0.645	1.78e-07	0.014	[38,39]
00600	Glycosphingolipid metabolism	32	7.84e-02	0.615	3.08e-07	0.016	[19]
00030	Pentose phosphate pathway	21	3.59e-07	0.022	2.80e-01	0.755	[38,39]
00062	Fatty acid elongation in mitochondria	11	1.68e-01	0.684	3.67e-07	0.022	[19,38]
00272	Cysteine metabolism	10	6.01e-07	0.025	7.52e-02	0.574	--
00340	Histidine metabolism	27	3.94e-02	0.477	1.42e-06	0.022	[39]
00720	Reductive carboxylate cycle	9	7.62e-02	0.574	1.51e-06	0.025	[19]
00565	Ether lipid metabolism	23	4.07e-06	0.036	8.43e-01	0.948	--
01032	Glycan structures - degradation	39	8.17e-01	0.957	4.62e-06	0.038	
00360	Phenylalanine metabolism	19	2.32e-02	0.376	6.26e-06	0.044	[38,39]
00040	Pentose and glucuronate interconversions	17	7.75e-06	0.047	4.98e-01	0.843	[19]
00051	Fructose and mannose metabolism	35	4.49e-03	0.211	7.99e-06	0.043	[19,38]

The *L_p _*column lists the size of the pathway. *χ*^2 ^test *p*-values for tumor status versus cluster assignment in PDM layer 1 and layer 2 are given. The *f*_rand _columns show the fraction of randomly-generated pathways with smaller *χ*^2 ^*p*-values in either PDM layer. The final column lists the data sets for which [29] identified the pathway as significant ([19], Singh; [38], Welsh; [39], Ernst; a dash indicates pathways with significant revisions (*>*30% of genes added or removed) in KEGG between this analysis and the time of [29] publication).

A number of known prostate cancer-related pathways appear at the top of this list. The urea acid cycle pathway, prion disease pathway, and bile acid synthesis pathways have previously been noted in relationship to prostate cancer [29]. The coagulation cascade is known to be involved in tumorigenesis through its role in angiogenesis [33], and portions of this pathway have been implicated in prostate metastasis [34]. Cytochrome P450, which is part of the inflammatory response, has been implicated in many cancers [35], including prostate [36], with the additional finding that it may play a role in estrogen metabolism (critical to certain prostate cancers) [37]. Many amino acid metabolism pathways (a hallmark of proliferating cells) and known cancer-associated signaling pathways (Jak-STAT, Wnt) are also identified.

Because Pathway-PDM does not rely upon single-gene associations and employs a "scrubbing" step to reveal progressively finer relationships, we expect that we will be able to identify pathways missed by other methods. It is of interest to compare the results obtained by Pathway-PDM to those obtained by other pathway analysis techniques. In [29], the authors applied several established pathway analyses (Fisher's test, GSEA, and the Global Test) to a suite of three prostate cancer gene expression data sets, including the Singh data considered here. Fifty-five KEGG pathways were identified in at least one data set by at least one method [29], but with poor concordance: 15 of these were found solely in the Singh data, and 13 were found in both the Singh data and at least one of the other two data sets (Welsh [38], Ernst [39]) using any method. A comparison of the Pathway-PDM identified pathways to those reported in [29] is given by the final column of Table 6, which lists the data sets for which that pathway was found to be significant using at least one method (Fisher's test, GSEA, and the Global Test) reported in [29]. Of the 29 Pathway-PDM identified pathways, 16 had been identified by [29] in either the Welsh or Ernst data (including 7 found by other methods in the Singh data by [29]). The PDM-identified pathways show improved concordance with the pathways identified in [29]; while only 13 of the 40 pathways identified in the Welsh or Ernst data were corroborated by the Singh data using any method in [29], the addition of the Pathway-PDM Singh results brings this to 22/40. Of the 13 pathways newly introduced in Table 6, several are already known to play a role in prostate cancer but were not detected using the methods in [29] (such as cytochrome P450, complement and coagulation cascades, and Jak-STAT signalling); several also constitute entries in KEGG that were either not present at the time that [29] was published or have had over 30% of genes added/removed, making them incomparable to the KEGG annotations used in [29]. This improved concordance supports the inferred role of the PDM-identified pathways in prostate cancer, and, as applied to the Singh data, suggests that the Pathway-PDM is able to detect pathway-based gene expression patterns missed by other methods.

## Conclusions

We have presented here a new application of the Partition Decoupling Method [14,15] to gene expression profiling data, demonstrating how it can be used to identify multi-scale relationships amongst samples using both the entire gene expression profiles and biologically-relevant gene subsets (pathways). By comparing the unsupervised groupings of samples to their phenotype, we use the PDM to infer pathways that play a role in disease.

The PDM has a number of features that make it preferable to existing microarray analysis techniques. First, the use of spectral clustering allows identification of clusters that are not necessarily separable by linear surfaces, enabling the identification of complex relationships between samples. As this relates to microarray data, this corresponds to the ability to identify clusters of samples even in situations where the genes do not exhibit differential expression. This is particularly useful when examining gene expression profiles of complex diseases, where single-gene etiologies are rare. We observe the benefit of this feature in the example of Figure 2, where the two separate yeast cell groups could not be separated using *k*-means clustering but could be correctly clustered using spectral clustering. We note that, like the genes in Figure 2, the oscillatory nature of many genes [28] makes detecting such patterns crucial.

Second, the PDM employs not only a low-dimensional embedding of the feature space, thus reducing noise (an important consideration when dealing with noisy microarray data), but also the optimal dimensionality and number of clusters is data-driven rather than heuristically set. This makes the PDM an entirely unsupervised method. Because those parameters are obtained with reference to a resampled null model, the PDM prevents samples from being clustered when the relationships amongst them are indistinguishable from noise. We observed the benefit of this feature in the radiation response data [18] shown in Figure 3, where two (as opposed to four) phenotype-related clusters were articulated by the PDM: the first corresponding to the high-RS cases, and the second corresponding to a combination of the three control groups.

Third, the independent "layers" of clusters (decoupled partitions) obtained in the PDM provide a natural means of teasing out variation due to experimental conditions, phenotypes, molecular subtypes, and non-clinically relevant heterogeneity. We observed this in the radiation response data [18], where the PDM identified the exposure groups with 100% accuracy in the first layer (Figure 3 and Table 2) followed by highly accurate classification of the high-RS samples in the second layer (Figure 3 and Table 5). The improved sensitivity to classify high-RS samples over linear methods (83% vs. the 64% reported using SAM in [18]) suggests that there may exist strong patterns, previously undetected, of gene expression that correlate with radiation exposure and cell type. This was also observed in the benchmark data sets [9], shown in Figure 4 and supplementary Figs. S-1, S-2 (Additional Files 1 and 2), where the PDM automatically detected subtypes in an unsupervised manner without forcing the cluster number. The results from the PDM in the radiation response data and benchmark data sets were at least as and generally more accurate than those reported using other algorithms in [9,18], were obtained without assumptions regarding the sample classes, and reflect statistically significant (with reference to the resampled null model) relationships between samples in the data.

The accuracy of the PDM can be used, in the context of gene subsets defined by pathways, to identify mechanisms that permit the partitioning of phenotypes. In Pathway-PDM, we subset the genes by pathway, apply the PDM, and then test whether the PDM cluster assignments reflect the known sample classes. Pathways that permit accurate partitioning by sample class contain genes with expression patterns that distinguish the classes, and may be inferred to play a role in the biological characteristics that distinguish the classes. This is a novel approach to pathway analysis that improves upon enrichment approaches in that does not require that the pathway's constituent genes be differentially expressed. That is, we expect that Pathway-PDM will identify both the pathways that would be identified in enrichment analyses (since differentially expressed genes imply linear cluster boundaries) as well as those whose constituent genes would not yield high measures of differential expression (such as in the two_circles example or the yeast cell-cycle genes). This makes Pathway-PDM a promising tool for identifying mechanisms that show systems-level differences in their regulation that could be missed by methods that rely on single-gene association statistics.

To illustrate Pathway-PDM, we applied the Pathway-PDM to both the radiation response data [18] and a prostate cancer data set [19]. In the radiation response data [18], we identified pathways that partitioned the samples by phenotype and both by phenotype and exposure (Figure 5) as well as pathways that only partitioned the samples by exposure without distinguishing the phenotypes (Figure S-3 in Additional File 3). In the prostate cancer data [19], we identified 29 pathways that partitioned the samples by tumor/normal status (Table 6). Of these, 15 revealed the significant tumor/normal partition in the second layer rather than the first (as did the full-genome PDM--see Figure S-4 in Additional File 4), and 13 of the 14 pathways with significant tumor/normal partitions in the first layer contained additional structure in the second. Prostate cancer is known to be molecularly diverse [19], and these partitions may reflect unidentified subcategories of cancer or some other heterogeneity amongst the patients. By applying the Pathway-PDM to the Singh data, we were able to improve upon the pathway-level concordance reported in [29], which applied pathway enrichment analyses (including GSEA) to data from the Singh, Welsh, and Ernst prostate cancer studies. We find not only that Pathway-PDM identifies pathways in the Singh prostate data that were identified in [29], but in addition identifies several other pathways from the Singh data that were reported by [29] in the Welsh and Ernst data, but not in the Singh data. That is, despite the fact that these pathways were not identified in the Singh data using GSEA, there do exist patterns of gene expression that are detected by Pathway-PDM; their identification in the other two data sets corroborates their relevance and supports their further investigation.

While our application of Pathway-PDM was such that the clusters found by the PDM for each pathway were compared against known sample class labels, we can just as easily compare them to labels from the cluster assignment from full-genome PDM. Hence, for example, in a situation such as the Golub-1999-v1 data shown in Figure 4(a), we could use the 3-cluster assignment, rather than the 2-class sample labels, to find the pathways that permit the separation of cluster-2 ALLs from the cluster-3 ALLs. In a case like this, where full-genome PDM analysis suggests the existence of disease subtypes, applying Pathway-PDM may help identify the molecular mechanisms that distinguish those samples. (Note that the use of the PDM's resampled null model implies that such phenotype subdivisions are statistically significant, rather than the result of an arbitrary cut of a dendrogram.) Such an analysis would enable a refined understanding of the molecular differences between the subtypes and suggest alternative mechanisms to investigate for diagnostic and therapeutic potential.

Despite these benefits, the PDM as applied here has two potential drawbacks. First, while we obtained accurate results from the PDM when setting *σ *= 1, the dependence upon this scaling parameter in Eq. 1 is a known issue in kernel-based methods, including spectral clustering and KPCA [21,22]. Methods to optimally select *σ *are actively being developed, and several adaptive procedures have been suggested (eg, [40]) that may allow for refined tuning of *σ*. Second, the low-dimensional nonlinear embedding of the data that makes spectral clustering and the PDM powerful also complicates the biological interpretation of the findings (in much the same way that clustering in principal component space might). Pathway-PDM serves to address this issue by leveraging expert knowledge to identify mechanisms associated with the phenotypes. Additionally, the nature of the embedding, which relies upon the geometric structure of all the samples, makes the classification of a new sample challenging. These issues might be addressed in several ways: experimentally, by investigation of the Pathway-PDM identified pathways (possibly after further subsetting the genes to subsets of the pathway) to yield a better biological understanding of the dynamics of the system that were "snapshot" in the gene expression data; statistically, by modeling the pathway genes using an approach such as [41] that explicitly accounts for oscillatory patterns (as seen in Figure 2) or such as [13] that accounts for the interaction structure of the pathway; or geometrically, by implementing an out-of-sample extension for the embedding as described in [42,43] that would allow a new sample to be classified against the PDM results of the known samples.

In sum, our findings illustrate the utility of the PDM in gene expression analysis and establish a new technique for pathway-based analysis of gene expression data that is able to articulate phenotype distinctions that arise from systems-level (rather than single-gene) differences. We expect this approach to be of use in future analysis of microarray data as a complement to existing techniques.

## Methods

### Implementation and Availability

The PDM as described above was implemented in R [44] and applied to the data sets below. Genes with missing expression values were excluded when computing the (Pearson) correlation *ρ_ij _*between samples. In the *l*-optimization step, 60 resamplings of the correlation coefficients were used to determine the dimension of the embedding *l*. In the clustering step, 30 *k*-means runs were performed, choosing the clustering yielding the smallest within-cluster sum of squares. An free, open-source R package to carry out the PDM is available for download from http://braun.tx0.org/PDM.

### Data

#### Radiation Response Data

These data come from a gene-expression profiling study of radiation toxicity designed to identify the determinants of adverse reaction to radiation therapy [18]. In this study, skin fibroblasts from 14 patients with high radiation sensitivity (High-RS) were collected and cultured, along with those from three control groups: 13 patients with low radiation-sensitivity (Low-RS), 15 healthy individuals, and 15 individuals with skin cancer. The cells were then subject to mock (M), ultraviolet (U) and ionizing (I) radiation exposures. As reported in [18], RNA from these 171 samples comprising four phenotypes and three treatments were hybridized to Affymetrix HGU95AV2 chips, providing gene expression data for each sample for 12615 unique probes. The microarray data was normalized using RMA [45]. The gene expression data is publicly available and was retrieved from the Gene Expression Omnibus [46] repository under record number GDS968.

#### DeSouto Multi-study Benchmark Data

These data comprise filtered gene expression levels from 21 cancer studies using Affymetrix microarrays along with associated class labels. The data were analyzed previously in [9], where several clustering methods were applied to compare algorithmic performance. The data were obtained from their original sources and subjected to filtering as described in [9]; we obtained the filtered sets as used in [9] and made available by the authors. This permits a direct comparison of the PDM results to those reported in [9].

#### Singh Prostate Data

These data come from a gene-expression profiling study of prostate cancer comprising 52 tumor samples (T) and 50 tumor-adjacent normal samples (N) from 52 men who had undergone radical prostatectomy [19]. RNA was hybridized to Affymetrix HGU95AV2 chips, providing gene expression data for each sample for 12615 unique probes. The microarray data CEL files were downloaded from the Broad Institute website and normalized using RMA [45].

#### Pathway annotation

The BioConductor [47] annotation packages hgu95av2.db, hgu95a.db, and KEGG.db were used to map Affymetrix probe IDs to KEGG pathways. Only KEGG pathways were investigated. A total of 203 KEGG pathways containing genes probed in the above data were identified.

## Authors' contributions

RB, GL, SP, and DR conceived of the study and participated in its design. RB wrote the R PDM implementation, performed the statistical analysis, and drafted the manuscript. DR helped to draft the manuscript. All authors read and approved the final manuscript.

## Supplementary Material

Additional File 1 **Figure S-1**. PDM classifications of deSouto benchmark set samples using a correlation-based distance metric (as described in methods). Click here for file

Additional File 2 **Figure S-2**. PDM classifications of deSouto benchmark set samples using a Euclidean distance metric. Click here for file

Additional File 3 **Figure S-3**. Pathway-PDM classifications of radiation response data for pathways that discriminate cells by radiation exposure but not by phenotype, suggesting that these mechanisms are intact across sample types. Exposure is indicated by shape ("M", mock; "U", UV; "I", IR), with phenotypes (healthy, skin cancer, low RS, high RS) indicated by color. The discriminatory pathways relate to DNA metabolism and cell death, as would be expected from radiation exposure. Click here for file

Additional File 4 **Figure S-4**. PDM results in first and second layers of the Singh prostate tumor data using all genes. The top two panels show the Fiedler vector values and clustering results, along with the Fiedler vector density, in the first and second layer; the bottom panel shows the combined classification results. The second layer, but not the first, discriminates the tumor samples. Click here for file
