# Supplementary figures and images for: Partition decoupling for multi-gene analysis of gene expression profiling data
Source: BMC Bioinformatics. 2011 Dec 30;12:497. doi: 10.1186/1471-2105-12-497 (PMC3276603; doi:10.1186/1471-2105-12-497)

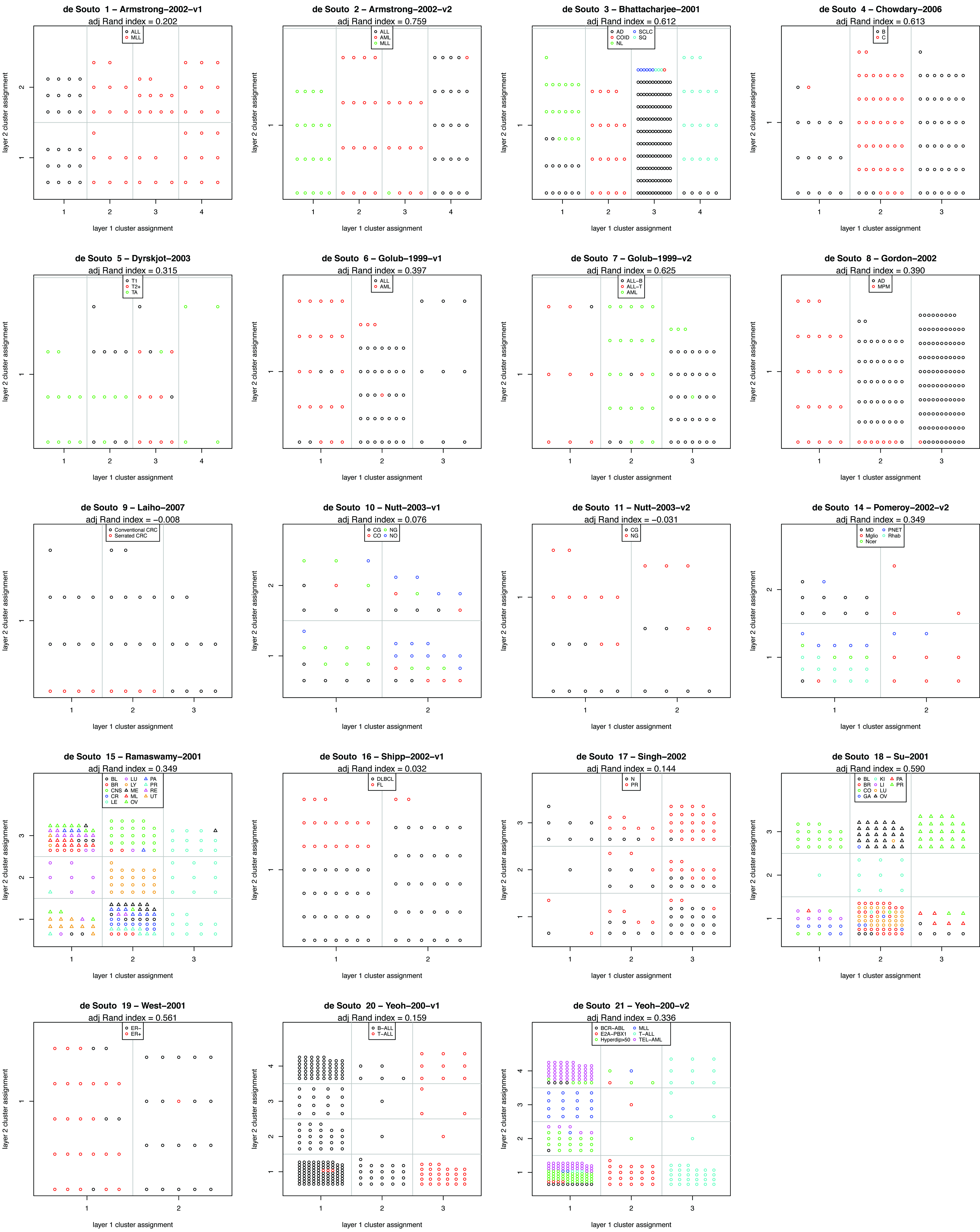

Supplement: Additional File 1 — Figure S-1. PDM classifications of deSouto benchmark set samples using a correlation-based distance metric (as described in methods). [file 1471-2105-12-497-S1.TIFF]

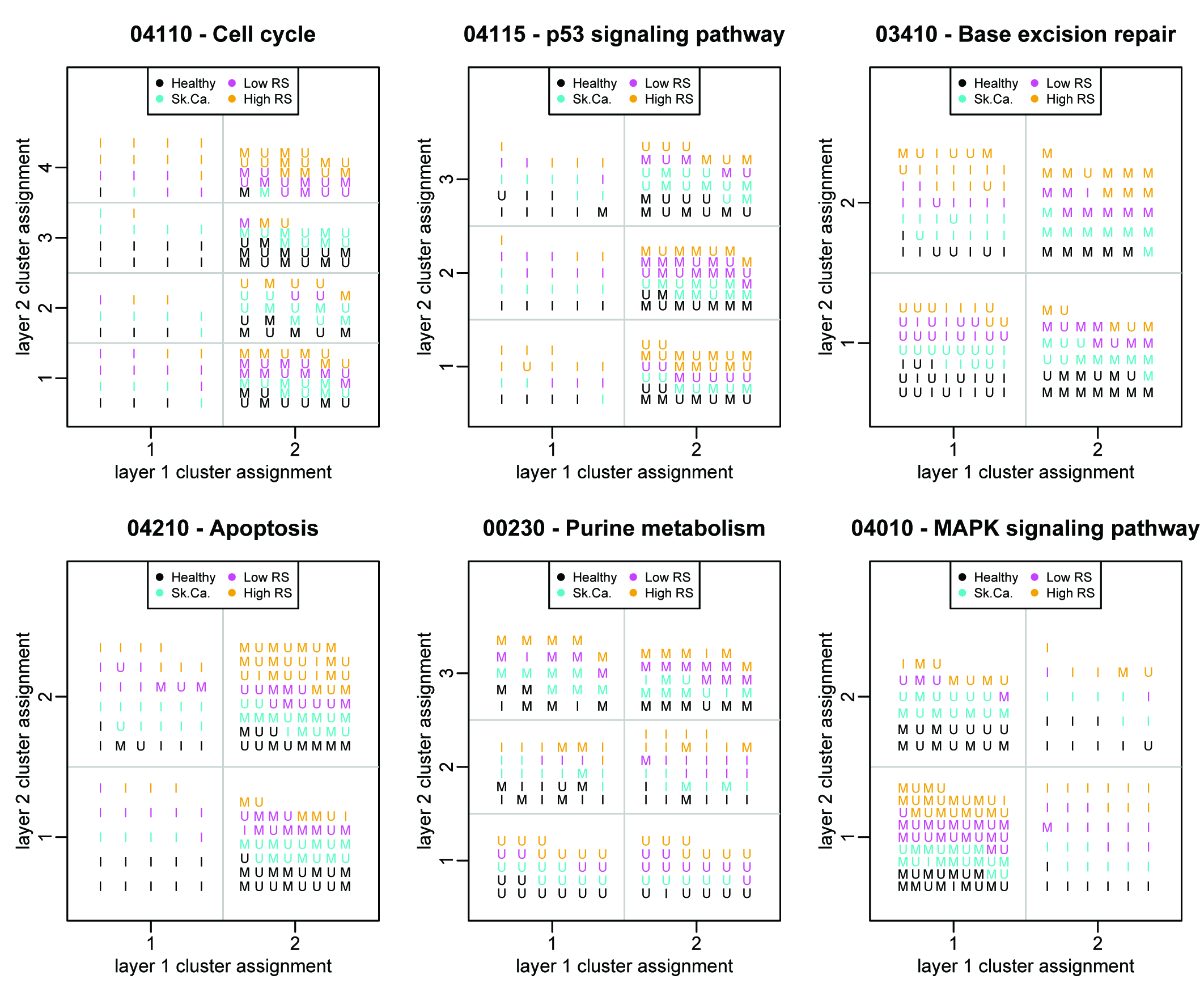

Supplement: Additional File 3 — Figure S-3. Pathway-PDM classifications of radiation response data for pathways that discriminate cells by radiation exposure but not by phenotype, suggesting that these mechanisms are intact across sample types. Exposure is indicated by shape ("M", mock; "U", UV; "I", IR), with phenotypes (healthy, skin cancer, low RS, high RS) indicated by color. The discriminatory pathways relate to DNA metabolism and cell death, as would be expected from radiation exposure. [file 1471-2105-12-497-S3.TIFF]

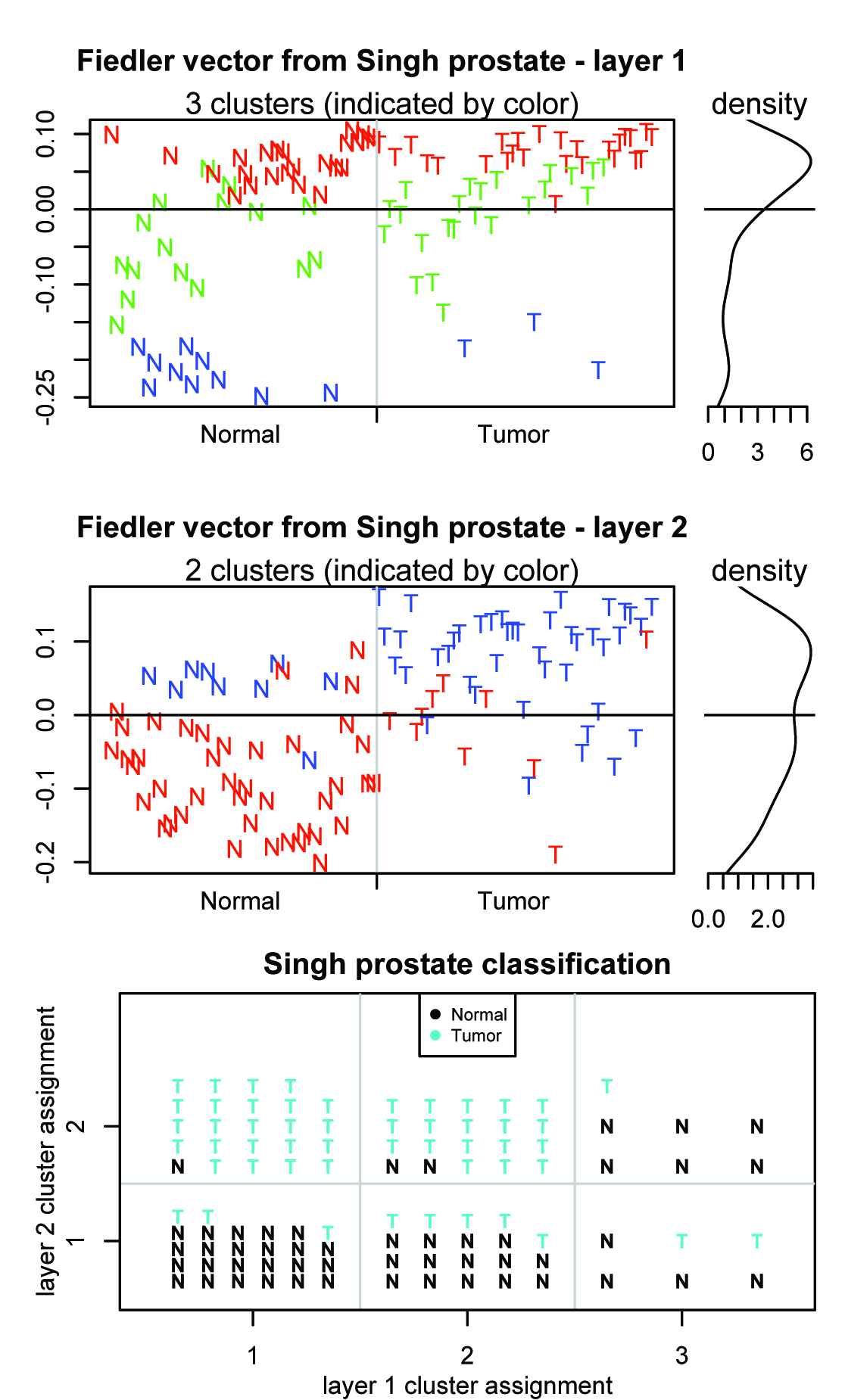

Supplement: Additional File 4 — Figure S-4. PDM results in first and second layers of the Singh prostate tumor data using all genes. The top two panels show the Fiedler vector values and clustering results, along with the Fiedler vector density, in the first and second layer; the bottom panel shows the combined classification results. The second layer, but not the first, discriminates the tumor samples. [file 1471-2105-12-497-S4.TIFF]
